# The first record of a shortnose chimaera-like egg capsule from the Mesozoic (Late Jurassic, Switzerland)

**DOI:** 10.1186/s13358-025-00352-x

**Published:** 2025-02-16

**Authors:** Yang Zhao, Jordan Bestwick, Jan Fischer, Dylan Bastiaans, Merle Greif, Christian Klug

**Affiliations:** 1 Department of Palaeontology, University of Zurich, 8006 Zurich, Switzerland; 2 Urweltmuseum GEOSKOP, Burg Lichtenberg (Pfalz), 66871 Thallichtenberg, Germany; 3 Centre Céramique, Natural History Museum Maastricht, 6211 KJ Maastricht, The Netherlands

**Keywords:** *Laffonia*, *Pseudocaudina*, Egg capsule, Holocephali, Chimaeridae, Late Jurassic

## Abstract

Chondrichthyan egg capsules, fossil and recent, have a taxonomical significance that can provide important insights into the occurrence and reproductive strategy of their producers. However, the rare occurrence of fossil capsules and their sometimes difficult identification hinder our understanding of their systematics and significance. *Laffonia* from the Late Jurassic of Switzerland and its probable junior synonym, *Pseudocaudina,* from the Late Jurassic lithographic limestones of southern Germany, have been interpreted in a variety of ways including as a fructification of a plant, a possible egg capsule of a shark or ray, a presumed holothurian, a possible actinarian, or even a ctenophore*.* Here, we redescribe the holotype of *Laffonia*, which has a fusiform body that is ornamented with over seven longitudinal ribs and two narrow striated flanges at its lateral edges. These morphological features are incompatible with a diploblast or echinoderm affinity, but highly resemble the characteristics of certain holocephalan egg capsules in several respects. Our phylogenetic analysis places *Laffonia* within a group containing the Carboniferous fossil capsules *Crookallia* and *Vetacapsula*, as well as recent chimaerid capsules. Thus, we suggest that the Mesozoic *Laffonia* represents an intermediate morphotype between the Carboniferous species and extant chimaerid capsules. *Laffonia* is the only known fossil chimaerid-like capsule from the Mesozoic so far, which offers novel insights into the morphology and evolution of holocephalan egg capsules.

**Supplementary Information:**

The online version contains supplementary material available at 10.1186/s13358-025-00352-x.

## Introduction

The Holocephali is a monophyletic group of Chondrichthyes (cartilaginous fishes) informally known as chimaeras and their extinct relatives (Didier et al., 2012; Inoue et al., 2010; Stahl, 1999). Extant holocephalans are represented by three families, Callorhynchidae (plownose chimaeras), Rhinochimaeridae (longnose chimaeras) and Chimaeridae (shortnose chimaeras), that likely diverged in the Mesozoic (Heinicke et al., 2009; Inoue et al., 2010; Licht et al., 2012). Together, these families comprise the subgroup Chimaeroidei (Didier, 1995; Didier et al., 2012; Patterson, 1965). All modern holocephalans are oviparous (egg-laying) producing tough, leathery capsules that are usually deposited on the ocean floor (Dean, 1906; Didier et al., 2012; Wourms, 1977). Each extant holocephalan egg capsule has a distinctive morphology that allows taxonomic identification to the family level (Dean, 1906; Didier, 1995; Didier et al., 2012; Fischer, 2018).

Fossil egg capsules can provide vital information not only on the capsules themselves, including their morphology and size, but also on the reproductive strategies of potential producers, such as their palaeodistribution and depositional settings (e.g., nursery grounds; Fischer et al., 2011, 2014; Gottfried & Fordyce, 2015; Sallan & Coates, 2014). However, some fossil capsules, such as *Palaeoxyris* and *Fayolia* (both elasmobranch capsules) as well as *Vetacapsula* (holocephalan capsule), were not correctly identified upon discovery and were initially described as plant remains (Crookall, 1932; Fischer & Kogan, 2008). In addition, the fossil record of egg capsules is sparse. In terms of holocephalan capsules, despite the discoveries of several fossil capsules produced by potential callorhynchids or rhinochimaerids, there previously was no convincing evidence of chimaerid egg capsules from the Mesozoic (Fischer et al., 2014; Stahl, 1999). Misidentification and their rare occurrence impede our understanding on the systematics and evolution of capsule producers, including holocephalans and other chondrichthyans.

*Laffonia* from the Late Jurassic of Switzerland is such a case of enigmatic fossil, which was initially compared to the fructification of a plant or an egg capsule of (non-holocephalan) chondrichthyans (Heer, 1877). However, insufficient understanding of morphological characters in *Laffonia* and its potential junior synonym *Pseudocaudina* has made the affinity uncertain for over 140 years, which has been variously identified as a diploblastic animal (actinarian or ctenophore) or an echinoderm (holothurian) (Broili, 1926; Conway Morris & Collins, 1996; Heding, 1932; Reich, 2015; Ziegler, 1991). Here, we redescribe the holotype of *Laffonia* and its morphological features. Our phylogenetic analysis places *Laffonia* in the systematics of the holocephalan capsules.

## Material and methods

**Fig. 1:**
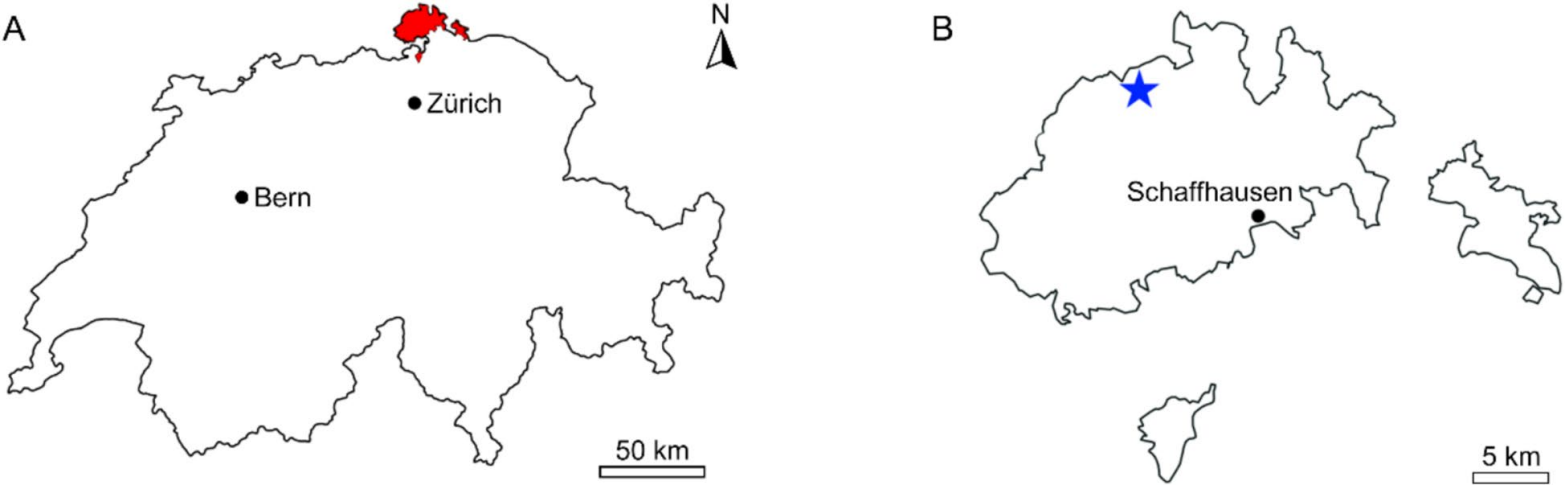
Map of the study area. **A** Location of the Canton of Schaffhausen in Switzerland. **B** the location of where *Laffonia* was found within the canton

**Fig. 2:**
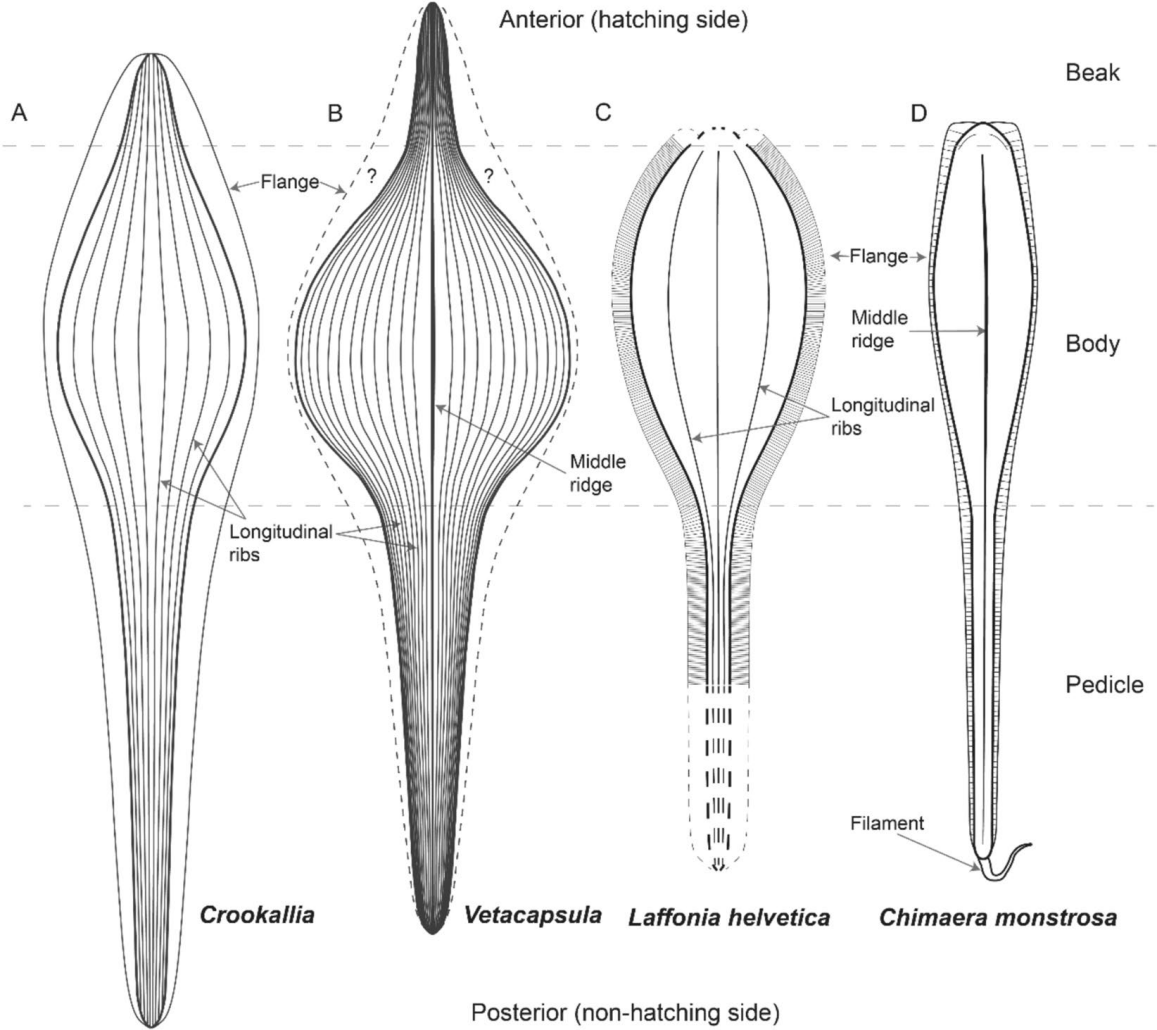
Schematic drawings of fossil and modern holocephalan egg capsule morphotypes. **A** *Crookallia,* **B** *Vetacapsula,* **C** *Laffonia helvetica,* and **D** *Chimaera monstrosa*. **A** and **B** are modified from Fischer et al. (2014) and Mottequin et al. (2022); **D** is modified from Mancusi et al. (2021). The very anterior and posterior edges of the *Laffonia* capsule are purely hypothetical. Drawings are not to scale

The specimen PIMUZ 5272 was discovered by Johann Conrad Laffon in a quarry near Beggingen, in the Canton of Schaffhausen, Switzerland (Fig. 1) and was formally erected as *Laffonia helvetica* by Oswald Heer in 1877. The specimen was originally described as from the Wangener-Schichten Member (= Wangen Member) of the Balsthal Formation (Heer, 1877) and was later described as from the upper Oxfordian *planula* ammonite zone (Ziegler, 1991). However, neither of these lithostratigraphic units is known from Schaffhausen, and the *planula* zone is lower Kimmeridgian (Gygi, 2000, 2013). Although we cannot be fully certain, the specimen instead likely originates from the Küssaberg Member of the Villigen Formation. These lithostratigraphic units occur in Schaffhausen and are contemporaneous with the Wangen Member and Balsthal Formation (Gygi, 2000, 2013). In addition, the Küssaberg and Wangen Members contain very similar lithologies (yellow-white micritic limestone beds; Gygi, 2000, 2013) which also supports our claim. The Küssaberg Member is tentatively assigned to the *Bimammatum* zone which is upper Oxfordian (Gygi, 2000, 2013) and so the most likely stratigraphic position of the specimen does not differ from the original description. The specimen PIMUZ 5272 is housed at the Department of Palaeontology of the University of Zurich. The fossil was photographed with a Nikon D2X camera and VHX-7000 digital microscope. The images were imported into Adobe Photoshop CC 2019 to remove the background and adjust contrast levels, brightness, and size. The schematic and interpretive drawings are based on these photos and the fossil material. To compare the size of *Laffonia* with other fossil and recent holocephalan egg capsules, capsule body lengths (in an anterior–posterior direction) and widths were measured from published images with scale bars in ImageJ 1.51j8. Capsule morphological terminology (Fig. 2) is adopted from Fischer et al. (2014) and Mottequin et al. (2022).

A phylogenetic dataset containing 22 chondrichthyan capsule characters of 16 taxa was modified from Fischer et al. (2014) (see supplementary information). Parsimony analyses were carried out in TNT 1.5 (Goloboff et al., 2008) with equal character weights. A traditional search with 10,000 replicates was performed using the swapping algorithm of tree bisection reconnection, with 10 trees saved per replication. A potential Placodermi egg capsule from the Devonian (Chaloner et al., 1980) was used as an outgroup. A strict consensus tree was calculated based on 10 parsimonious trees. The support values at nodes of the consensus tree were generated from 10,000 replicates of bootstrap and jackknife as well as Bremer support.

### Systematic palaeontology

Ichnogenus *Laffonia* Heer, 1877 (= *Pseudocaudina* Broili, 1926)

Diagnosis. A fusiform egg capsule with an elongated pedicle. The capsule is divided into dorsal and ventral sides by narrow lateral striated flanges. The dorsal and lateral surfaces of the body are ornamented with possibly up to nine longitudinal ribs.

Occurrence. Most likely upper Oxfordian, Late Jurassic. The specimen was most likely from the Küssaberg Member of the Villigen Formation, *Bimammatum* zone.

Holotype. PIMUZ 5272, a three-dimensional and incomplete internal mould preserved in a yellowish white limestone, missing the inferred anterior end of the beak and the distal end of the pedicle.

Other material. *Pseudocaudina* (see the holotype in Broili, 1926 and specimen JME SOS4372 in Reich, 2015).

**Remarks**

*Laffonia* Heer, 1877, highly resembles *Pseudocaudina* Broili, 1926, in having a fusiform body that is ornamented with several longitudinal ribs, although the latter is intensely compacted into a flat impression (Broili, 1926). In both taxa, the inferred posterior body end tapers to a narrow tail-like appendage (i.e. pedicle), while the anterior end of the capsule is broken. *Pseudocaudina* was found with half a dozen specimens (Reich, 2015) in Tithonian platy limestones (lithographic limestone) of the Upper Jurassic (Broili, 1926) of the Langenaltheim, Eichstätt and Solnhofen region. Thus, it is slightly younger than the only known *Laffonia* specimen. Considering the similarities in body shape, size, and morphological characters (e.g. longitudinal ribs), the small age difference (less than 10 My) and geographical proximity to each other, identifying *Pseudocaudina* as a junior synonym for *Laffonia* (Reich, 2015; Ziegler, 1991) is justified.

**Fig. 3:**
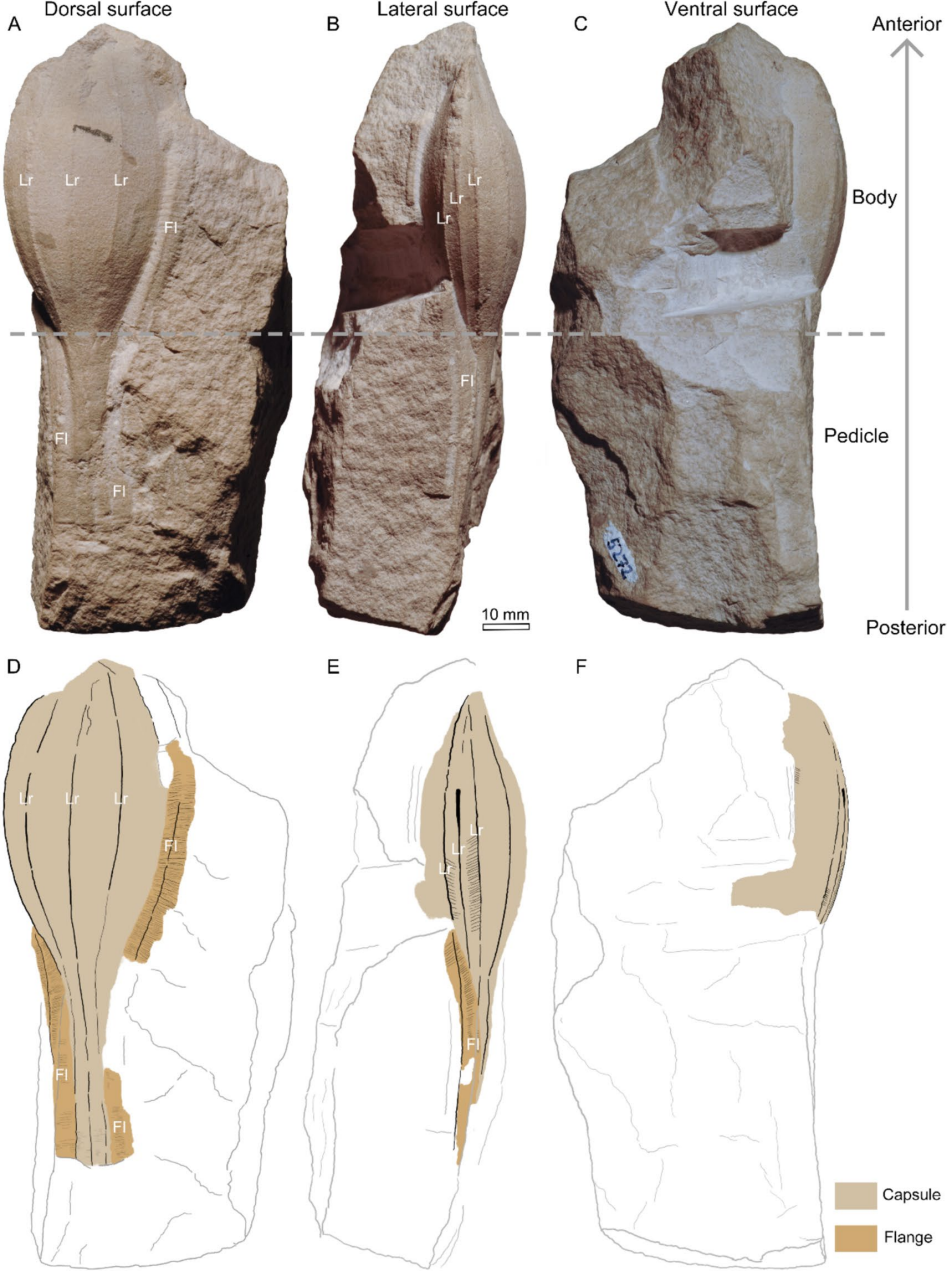
Holotype of *Laffonia helvetica* (PIMUZ 5272). **A**–**C** Inferred dorsal, lateral and ventral view, respectively. **D**–**F** interpretative drawings of (**A**–**C**), respectively. Fl: flange; Lr: longitudinal rib

**Fig. 4:**
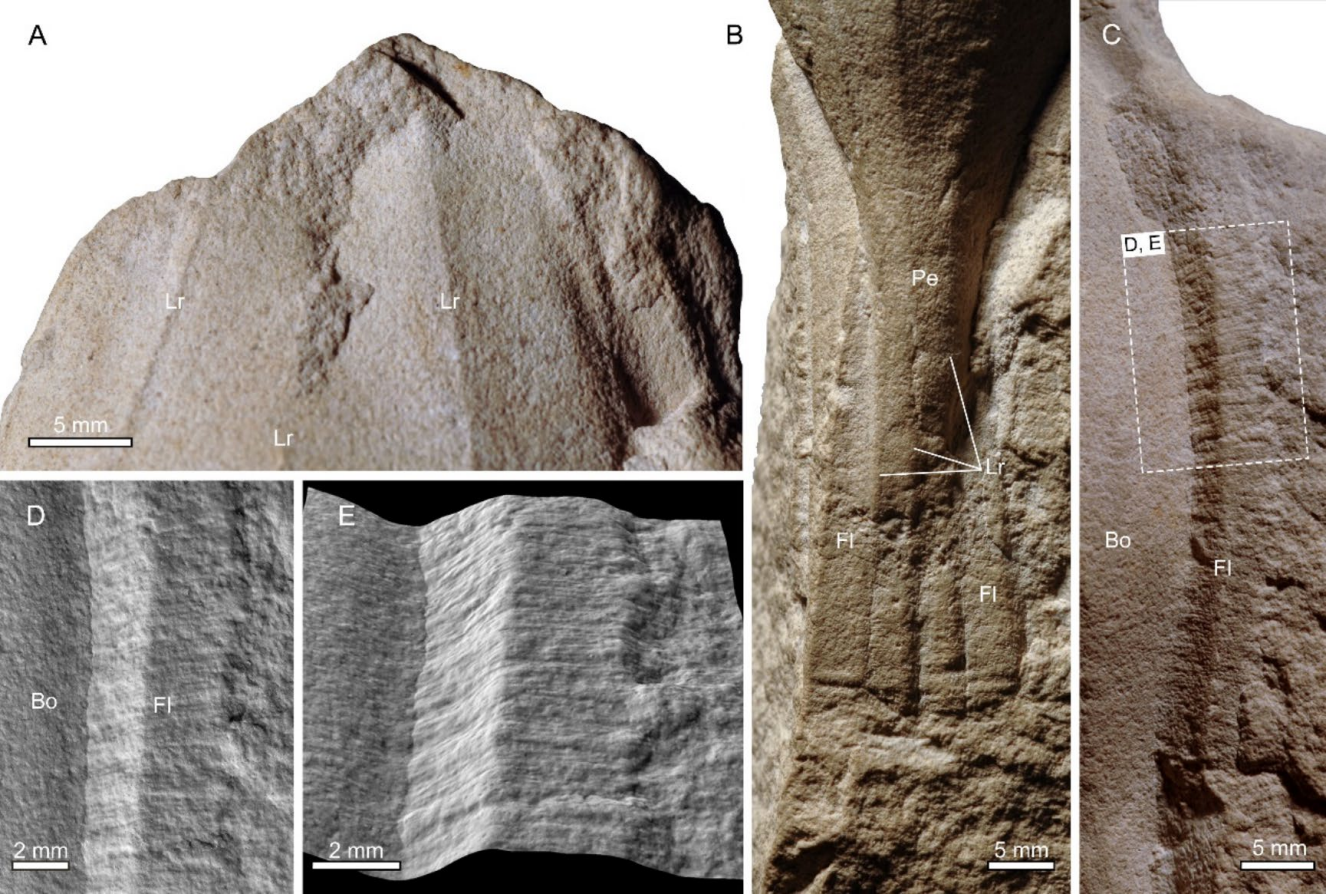
Details of the holotype of *Laffonia helvetica* (PIMUZ 5272). **A** anterior part of the body. **B** posterior part of the body, showing an elongated pedicle accompanied by two lateral flanges. **C** a lateral flange along the right side of the capsule body. **D–E** optical shadow effect mode images of the boxed region in (**C**), showing the flange ornamented with dense striations. Bo: body; Fl: flange; Lr: longitudinal rib; Pe: pedicle

*Laffonia helvetica* Heer, 1877

(Figs. 3, 4; see also the surface scan data on Sketchfab)

**Description**

The specimen exhibits a three-dimensionally preserved fusiform capsule (Fig. 3A–C). It measures round to 108 mm in length, 41 mm in width across the middle of the inferred dorsal surface, and 7 mm in maximum height in the lateral body surface. The central body is about 67 mm long and 35 mm wide. It tapers distally with the inferred anterior end slightly wider than the posterior. The anterior end of the body is truncated and not preserved, and the body surface herein is slightly folded inwards (Figs. 3A and 4A), while the posterior extends outwards forming a long, narrow appendage, i.e. the pedicle (Figs. 3A, B and 4B). The preserved part of the pedicle is straight and gradually flattens and fades into the rock without a clear end (Figs. 3A, 4B). The width of the distal-most preserved edge of the pedicle is 7 mm and the length of the pedicle is around 40 mm.

The body surface is ornamented with at least seven distinctive longitudinal ribs (Figs. 3A, B, 4A, B) that are interrupted at the broken anterior end but continue along the pedicle until its preserved distal-most edge. Three ribs are evenly distributed on the dorsal surface (Fig. 3A, D), and the distance between the ribs on the middle region is about 11 mm. Three ribs are arranged densely on the inferred left lateral surface (Fig. 3B, E), with interval distance being 3–4 mm. No longitudinal ribs are identified on the ventral surface, which is largely obscured by the matrix (Fig. 3C, F). The right lateral surface is only partially exposed at the anterior end of the body, which is apparently compacted into a flat plane (Fig. 3A). Numerous fine oblique lines that are arranged longitudinally are visible between two ribs on the lower middle of the left lateral surface (Fig. 3B, E), but no such lines are observed on the dorsal surface.

A narrow band of about 5 mm wide and at least 45 mm long extends along the right edge of the central body (Figs. 3A, D and 4C). It is interrupted at the upper lateral side of the pedicle, and the same band continues along the lower lateral side of the pedicle (Figs. 3A, D and 4B). The narrow band contains dense transverse striations and exhibits a prominent longitudinal ridge along the midline of the band (Fig. 4C–E). Another such narrow band is also visible at the left edge of the capsule, which extends along the entire length of the pedicle, but it is interrupted at the lower left side of the body and is slightly bent towards the ventral side (Figs. 3A, B, D, E and 4B). The narrow bands are here interpreted as flanges. Overall, it is likely that the flange was present in life along the entire lateral body edges.

### Phylogenetic results

**Fig. 5:**
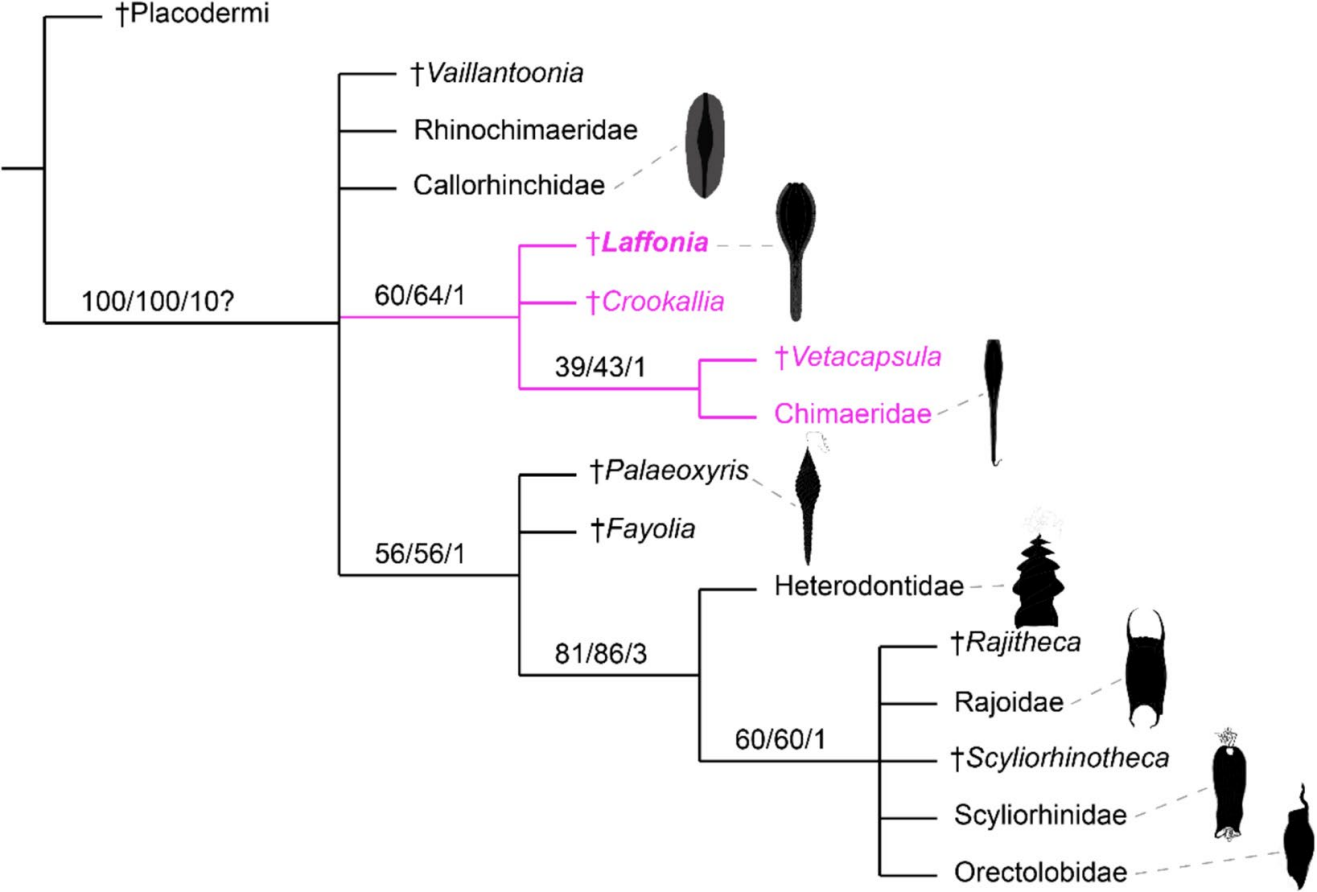
Phylogenetic relationship of chondrichthyan egg capsules. *Laffonia* is placed in a group (coloured pink) that comprises Carboniferous capsule types and extant chimaerid capsules. Numbers at nodes are support values from bootstrap/jackknife/Bremer. Fossil taxa are indicated by the dagger symbol

The consensus tree places *Laffonia* in a clade consisting of *Crookallia*, *Vetacapsula,* and recent chimaerid egg capsules, and the latter two branches form a sister group (Fig. 5). This group is supported by bootstrap and jackknife values of 60% and 64%, respectively. Recent elasmobranch egg capsules and the fossil capsules *Palaeoxyris*, *Fayolia*, *Scyliorhinotheca* and *Rajitheca* are resolved as a clade as well, with *Palaeoxyris* and *Fayolia* at the most basal position of this clade (Fig. 5). These two clades are placed in a polytomy with rhinochimaerid and callorhinchid egg capsules and *Vaillantoonia* (Fig. 5).

## Discussion

### *Laffonia* is neither a ctenophore, an actinarian nor a holothurian

The affinity of *Laffonia* has never been clarified unequivocally since its discovery (Heer, 1877; Reich, 2015; Ziegler, 1991). *Laffonia* and its probable junior synonym *Pseudocaudina* were described as morphologically resembling extant diploblastic animals including ctenophores (comb jelly) and anthozoans (sea anemones). *Laffonia* was most recently regarded as the closest to the Beroida, a ctenophore group that lacks a pair of tentacles (Ziegler, 1991). The presumed similarity is based mainly on the presence of striated bands in *Laffonia,* which supposedly are reminiscent of the cilia-bearing comb rows in extant ctenophores (Ziegler, 1991). However, the identification of comb rows in *Laffonia* is only tentative at best (Conway Morris & Collins, 1996). Ctenophore comb rows are composed of numerous independent ctenes that are serially arranged on the external body surface along an oral-aboral axis and each ctene includes a basal cushion plate that carries numerous macrocilia used for swimming (Tamm, 2014). The striated bands of *Laffonia* are only visible along the inferred lateral edges of the capsule, indicating they are apparently restricted to this region. The transverse striations are densely arranged along the band with no distinctly raised bars (Fig. 4D, E), which is inconsistent with the cushion plates of extant ctenophores or the cushion-like structures in well-preserved ctenophore fossils (Conway Morris & Collins, 1996; Ou et al., 2015; Parry et al., 2021; Stanley & Stürmer, 1983, 1987; Zhao et al., 2019). In addition, *Pseudocaudina* was once compared to an actinarian based on potential similarities in the column-like body (Heding, 1932). However, the presence of lateral striated bands and the lack of reliable anthozoan characters such as mesentery, pedal disc, and circumoral tentacles in *Laffonia* and *Pseudocaudina* make this inference suspect.

*Pseudocaudina* was initially interpreted as a holothurian (Broili, 1926). The transverse striations visible at the inferred anterior end of *Pseudocaudina* were interpreted as the transverse muscles that presumably surround the body with no interruption at the inferred ambulacra, which is a typical characteristic of synaptid holothuroids (Synaptidae) (Broili, 1926). While a holothurian identification was subsequently cited several times following its description (Croneis & McCormack, 1932; Frizzell & Exline, 1966), this assignment was nevertheless still disputed (Heding, 1932; Hess, 1973; Reich, 2015; Smirnov, 2012; Ziegler, 1991). The suggested muscle system in *Pseudocaudina* is questionable for several reasons. The appearance of this system was likely created by the compression of its dorsal and ventral sides during fossilisation and is inconsistent with the preservation in the three-dimensionally preserved *Laffonia* (Ziegler, 1991). Neither *Pseudocaudina* nor *Laffonia* exhibit other diagnostic traits of holothurians such as a perioral ring of calcareous sclerites at their anterior ends, sclerites in their dermis, and radially arranged ambulacra (Reich, 2015). *Laffonia* and *Pseudocaudina* can therefore not be assigned to holothurians.

### *Laffonia* as a holocephalan egg capsule

*Laffonia* is here identified as having a fusiform ribbed capsule accompanied by two narrow striated flanges laterally, resembling a chondrichthyan egg capsule, as already noted by Heer (1877). At least ten capsule morphotypes are recognized among extant and extinct chondrichthyans, relating to the shape and ornamentation of the capsule, the number and morphology of the anterior and posterior appendages (beaks, horns, pedicles etc.) and the size and arrangement of the flange (lateral, spiral etc.; Fischer et al., 2014; Mancusi et al., 2021). *Laffonia* lacks the spirally twisted flanges typical of *Palaeoxyris* and *Fayolia*, two morphotypes (named after the respective genera) known only from the fossil record, with their producers most likely being extinct hybodontiform and xenacanthiform sharks, respectively (Fischer & Kogan, 2008; Fischer et al., 2011, 2014). The lack of spiral flanges also differs from extant heterodontid shark capsules (Fischer et al., 2014). The single elongated pedicle of *Laffonia* greatly differs from the pair of curved horns exhibited by egg capsules from extant and extinct batoids (rays), orectolobiformes (carpet sharks), and scyliorhinids (catsharks), and these three groups often exhibit extremely reduced flanges that are much narrower than that of *Laffonia*. (Caruso & Bor, 2007; Concha et al., 2010; Fischer et al., 2014; Kiel et al., 2011; Mancusi et al., 2021; Steininger, 1966; Treloar et al., 2006). The morphology of *Laffonia* is therefore inconsistent with all currently recognised elasmobranch capsule morphotypes (Ziegler, 1991) and the specimen is thus unlikely to have been produced by an elasmobranch.

**Fig. 6:**
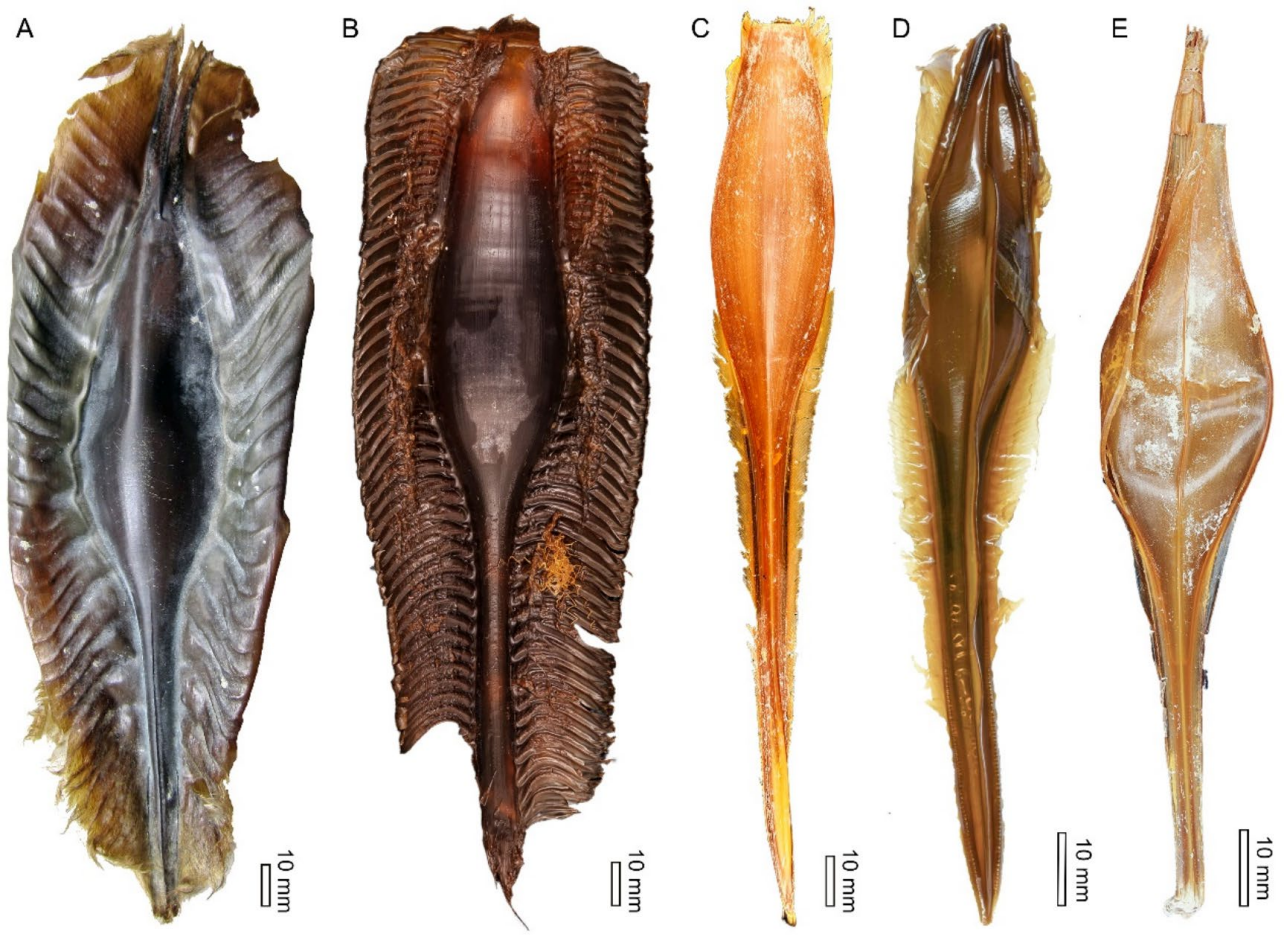
Modern holocephalan egg capsules. **A** *Callorhinchus milli* (EMRG-Chond-E-2), dorsal view. **B** *Rhinochimaera atlantica* (ZMH-ICH-123083), dorsal view. **C** *Chimaera monstrosa* (ZMH-ICH-101805), ventral view. **D** *Hydrolagus colliei* (EMRG-Chond-E-1), dorsal view. **E** *Hydrolagus mirabilis* (ZMH-ICH-122227), dorsal view. **A**, **D** Courtesy of Jürgen Kriwet and Lilith Mathiaschitz (Department of Palaeontology, University of Vienna). **B**, **C**, **E** courtesy of Thilo Weddehage (Leibniz Institute for the Analysis of Biodiversity Change, Hamburg, Germany)

In contrast to elasmobranch capsules, holocephalan capsule morphotypes exhibit relatively constrained morphologies from both extant and extinct taxa from across all three modern chimaeroid families (Fig. 6; Dean, 1906; Didier, 1995; Fischer et al., 2014). This morphotype is typically characterised by a fusiform capsule with a beak and pedicle that is all surrounded by laterally striated flanges that often vary in size between taxa (Fig. 6; Dean, 1906; Fischer et al., 2014). Comparable features between *Laffonia*, *Pseudocaudina* and the recent holocephalan morphotype include a fusiform capsule, pedicle and lateral flanges, although the incomplete preservation of the studied specimen prevents comparisons of a potential beak. More specifically, *Laffonia* exhibits several similarities to extant chimaerid capsules such as narrow lateral flanges, ribbed capsule surface and fine oblique lines like that of *Hydrolagus* (Fig. 6C–E; Dean, 1903). However, the flanges of *Laffonia* appear to be of equal size along the entire length of the capsule edges and possess a prominent longitudinal ridge. In contrast, the flanges of extant chimaerid capsules are relatively broad along the edges of the anterior and posterior capsule and reduce at the central body sides, with no obvious ridge (Fig. 6C–E; Dean, 1906; Mancusi et al., 2021). In addition, the at least seven longitudinal ribs that *Laffonia* exhibits on its inferred dorsal and lateral surfaces are not observed in recent holocephalan capsules (Fig. 6; Berio et al., 2024; Dean, 1906; Mancusi et al., 2021). This body ornamentation is more similar to that of the two Lower Pennsylvanian capsules, *Vetacapsula* and *Crookallia*, both of which are phylogenetically recovered as most closely related to chimaerid holocephalan morphotype (Fischer et al., 2014). Both taxa have ribbed capsules that are suggested to possess two narrow lateral flanges, although such flanges have not yet been documented in *Vetacapsula* (Fig. 2; Fischer & Kogan, 2008; Mottequin et al., 2022). However, the number of longitudinal ribs on the capsule surface is different among these three taxa, with over twenty ribs suggested in *Vetacapsula* (Crookall, 1928; Fischer & Kogan, 2008) and between eight and twenty in *Crookallia* (Mottequin et al., 2022). *Laffonia* and *Crookallia* lack a prominent middle ridge (dorsal keel) that is obvious in *Vetacapsula* and extant chimaerid capsules (Figs. 2, 6C–E; Fischer et al., 2014). A pedicle length exceeding body length is known from *Vetacapsula*, *Crookallia* and recent holocephalan capsules, but the incomplete preservation of the *Laffonia* pedicle prevents us from determining whether the Jurassic fossil shared this trait. In contrast to the Pennsylvanian fossil and modern chimaerid capsules, *Laffonia* possesses a larger central body and its body length–width ratio is closer to that of recent rhinochimaerid capsules than to any other holocephalan group (Table 1).

**Table 1:** Central body measurements and ratios of selected recent and fossil holocephalan egg capsules

Egg capsule	Nr	Producer	Age	L	W	L/W	References
*Vetacapsula cooperi*	FMNH PF 8961	Holocephalan	Pennsylvanian	25	21	1.19	McGhee and Richardson (1982)
*Crookallia debildei*	RBINS a7769	Holocephalan	Pennsylvanian	12	4	3	Mottequin et al. (2022)
*Crookallia kidstoni*	RBINS a13818	Holocephalan	Pennsylvanian	10	6	1.67	Mottequin et al. (2022)
*Crookallia preati*	n/a	Holocephalan	Pennsylvanian	12	5	2.4	Mottequin et al. (2022)
*Laffonia helvetica*	PIMUZ 5272	Chimaerid-like	Late Jurassic	67	35	1.91	This study
*Chimaera monstrosa*	n/a	Chimaerid	Recent	29	13	2.23	Mancusi et al. (2021)
*Hydrolagus colliei*	n/a	Chimaerid	Recent	23	10	2.3	Didier et al. (2012)
*Hydrolagus colliei*	EMRG-Chond-E-1	Chimaerid	Recent	44	17	2.59	Figure 6D
*Hydrolagus mirabilis*	ZMH-ICH-122227	Chimaerid	Recent	46	22	2.09	Figure 6E
*Vaillantoonia reperepe*	OU 22689	Callorhynchid	Late Triassic	> 56	24	> 2.33	Gottfried and Fordyce (2015)
*Vaillantoonia germanica*	SMNS 5043	Callorhynchid	Middle Jurassic	66	31	2.13	Böttcher (2010)
*Vaillantoonia schernfeldensis*	NHMUK PV Z.183	Callorhynchid	Late Jurassic	80	37	2.16	Duffin et al. (2022)
*Vaillantoonia schernfeldensis*	Frickhinger’s specimen	Callorhynchid	Late Jurassic	84	34	2.47	Duffin et al. (2022)
*Vaillantoonia schernfeldensis*	LF 703 & LF 5339^a^	Callorhynchid	Late Jurassic	80	32	2.5	Duffin et al. (2022)
*Callorhinchus milii*	n/a	Callorhynchid	Recent	29	14	2.07	Didier et al. (2012)
*Callorhinchus milli*	EMRG-Chond-E-2	Callorhynchid	Recent	86	34	2.53	Figure 6A
*Vaillantoonia arenicola*	92/1	Rhinochimaerid	Late Triassic	30	15	2	Harrison et al., 2021
*Vaillantoonia caucasica*	NMKBR IKP-25	Rhinochimaerid	Early Cretaceous	51	23	2.22	Harrison et al. (2021)
*Vaillantoonia gilli*	USNM 5994	Rhinochimaerid	Late Cretaceous	60	27	2.22	Harrison et al. (2021)
*Vaillantoonia gilli*	MEVE 1035	Rhinochimaerid	Late Cretaceous	54	26	2.08	Harrison et al. (2021)
*Vaillantoonia newmexicana*	USNM 16889	Rhinochimaerid	Late Cretaceous	84	33	2.55	Harrison et al. (2021)
*Rhinochimaera atlantica*	n/a	Rhinochimaerid	Recent	20	11	1.82	Didier et al. (2012)
*Rhinochimaera atlantica*	n/a	Rhinochimaerid	Recent	75	42	1.79	Chembian (2007)
*Rhinochimaera atlantica*	ZMH-ICH-123083	Rhinochimaerid	Recent	56	30	1.87	Figure 6B

All measurements rounded to the nearest mm. All ratios to 3 s.f

Nr.: specimen collection number; L: capsule body length; W: capsule body width; L/W: length–width ratio.

^a^LF 703 and LF 5339 are the part and counterpart of the same specimen, respectively

The morphological similarities between *Laffonia*, the Pennsylvanian fossil capsules and modern chimaerid capsules are supported by our phylogenetic analysis (Fig. 5). The unresolved polytomy relationships are likely due to the low number of characters and the incomplete preservation of the *Laffonia* capsule. Discovering additional material in the future may help to further clarify the phylogenetic position of *Laffonia*.

### Evolutionary significance

**Fig. 7:**
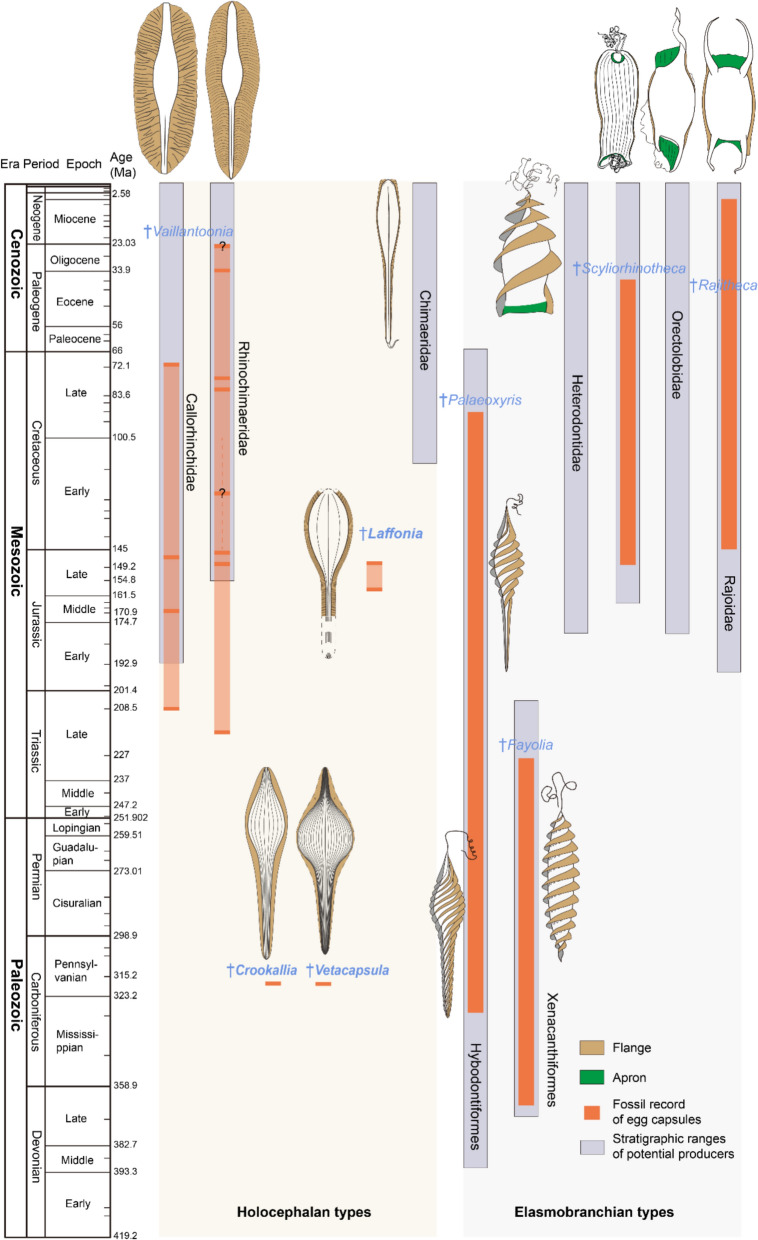
Stratigraphic distribution of chondrichthyan fossil egg capsule morphotypes and their potential producers, adapted from Fischer et al., 2013, 2014, 2023. Orange bars in Holocephali denote each known occurrence of capsules in the fossil record. All schematic drawings are not to scale

*Crookallia* and *Vetacapsula* from the Carboniferous (Pennsylvanian) supposedly represent the oldest records of capsules that were produced by holocephalans (Fischer et al., 2014; Mottequin et al., 2022), although there is a possibility that fossil capsules from the Middle or Late Devonian (Carr & Jackson, 2018; Chaloner et al., 1980) that have been regarded as placoderms could actually be from holocephalans (Fischer et al., 2014). However, the absence of transitional forms in both morphological and stratigraphical contexts makes these assignments tentative. The most definite holocephalan capsules are known from the Late Triassic of New Zealand (Gottfried & Fordyce, 2015) and Yakutia, Russia (Vozin, 1968), while most fossil capsules stem from the Jurassic and Cretaceous and few remains from the Paleogene (Fig. 7; Brown, 1946; Warren, 1948; Obruchev, 1967; Harrison et al., 2021; Duffin et al., 2022; Kiel et al., 2024; Johns et al., in press). Nevertheless, all currently known Mesozoic fossil capsules exhibit fusiform bodies with broad lateral flanges that greatly resemble either modern callorhinchid or rhinochimaerid capsules (Brown, 1946; Duffin et al., 2022; Gottfried & Fordyce, 2015; Harrison et al., 2021; Obruchev, 1967; Stahl, 1999; Vozin, 1968; Warren, 1948). Until now, 12 recognised species have been assigned to the ichnogenus *Vaillantoonia* Meunier, 1891, previously used under the generic name *Chimaerotheca* Brown, 1946 (Kiel et al., 2024), given that the convergence of capsule morphologies and the uncertainty of producers make it difficult to confidently assign them into specific extant genera, although such attempts were proposed in several studies (Obruchev, 1967; Stahl, 1999; Vozin, 1968). In contrast, no fossil capsule that possesses morphological features similar to either chimaerid capsules or Carboniferous taxa has been found in the Mesozoic, resulting in a long and uncertain ghost lineage (Fischer et al., 2014), although potential chimaerid body fossils go back at least to the Cretaceous (Duffin, 2001).

*Laffonia* and *Pseudocaudina* represent the first example of a chimaerid-like capsule from the Mesozoic, increasing the morphological diversity of known Mesozoic egg capsule morphotypes (Fig. 7). The reassignment of *Laffonia* not only supports the hypothesis that *Vetacapsula* and *Crookallia* were produced by holocephalans instead of elasmobranchs (Fischer et al., 2014), but that *Laffonia* most likely represents a transitional form between known Carboniferous morphotypes and extant chimaerid capsules. The capsule surfaces in *Laffonia, Crookallia* and *Vetacapsula* are all ornamented with several longitudinal ribs, indicating a ribbed capsule surface that likely represents an ancestral state of the total-group chimaerid egg capsules. Compared to the Carboniferous taxa, the number of longitudinal ribs has been reduced in Jurassic *Laffonia*, and only one prominent dorsal middle ridge is retained in extant chimaerid capsules*.* However, considering a similar middle ridge is possibly present in *Vetacapsula*, it is uncertain whether the prominent middle ridge is a plesiomorphic or a derived feature from the longitudinal ribs until more chimaerid fossil capsules are revealed. A narrow lateral flange is present both in *Crookallia* and *Laffonia.* However, the flange surface is likely smooth in *Crookallia* while it is striated in *Laffonia*. They are different from the ribbed flange in extant chimaerid capsules, indicating that the modern narrow flange may have appeared even later. More broadly, the morphological differences between fossil and recent capsules also indicate changes in holocephalan body size evolution even in the absence of body fossils. For example, it is reported in modern holocephalans that total capsule length is around one fifth to one quarter the body length of the producer (Dean, 1904, 1905). Assuming similar relationships for extinct taxa, it is reasonable to suggest that *Laffonia* was produced by a larger individual than the producers of the Carboniferous capsules, indicating an increase in body size of chimaerid-like holocephalans between the Carboniferous and Jurassic. However, the possibility that the Carboniferous capsules were produced by sexually mature, but still skeletally immature individuals cannot be ruled out, nor can the possibility of preservation bias in the Carboniferous in favour of smaller capsules.

## Conclusion

Our redescription of the holotype of *Laffonia* (and the suggested junior synonym *Pseudocaudina*) and our analyses suggest that it is a holocephalan egg capsule with morphological characters largely comparable to chimaerid capsules, rather than a ctenophore, actinarian or holothurian. This reassignment is supported by our phylogenetic analysis. It suggests that *Laffonia* represents the first fossil capsule from the Mesozoic potentially produced by a chimaerid or chimaerid-like animal. *Laffonia* possesses a fusiform body with a reduced number of longitudinal ribs and narrow, laterally striated flanges, indicating that it represents an intermediate morphotype between Carboniferous *Crookallia* and *Vetacapsula* and extant chimaerid capsules. However, their precise affinity will remain uncertain until further material is discovered and described.

## Supplementary Information

Additional file 1.

## Data Availability

Phylogenetic information and matrix are available in the supplementary material. Surface scan data of *Laffonia* are available via the following link: https://sketchfab.com/3d-models/laffonia-helvetica-pimuz-5272-20fd918d64914432be88baadef52c81e.

## Abbreviations

EMRG: Collection of the Evolutionary Morphology Research Group at the Department of Palaeontology, University of Vienna, Vienna, Austria
FMNH: Field Museum of Natural History, Chicago, Illinois, U.S.A.
JME: Jura-Museum Eichstätt, Eichstätt, Germany
LF: Lauer Foundation for Paleontology, Science and Education, Illinois, U.S.A.
MEVE: Mesa Verde National Park, Colorado, U.S.A.
NMKBR: National Museum of the Kabardino-Balkarian Republic, Nalchik, Russia
NHMUK: The Natural History Museum, London, U.K.
OU: Geology Museum, University of Otago, Dundein, New Zealand
PIMUZ: Paläontologisches Institut, Universität Zürich, Zürich, Switzerland
RBINS: Royal Belgian Institute of Natural Sciences, Brussels, Belgium
SMNS: Staatliches Museum für Naturkunde Stuttgart, Stuttgart, Germany
USNM: National Museum of Natural History, Washington D.C., U.S.A.
ZMH: Leibniz Institute for the Analysis of Biodiversity Change, Hamburg, Germany
