# Supplementary material for: The first record of a shortnose chimaera-like egg capsule from the Mesozoic (Late Jurassic, Switzerland)
Source: Swiss J Palaeontol. 2025 Feb 16;144(1):8. doi: 10.1186/s13358-025-00352-x (PMC11830639; doi:10.1186/s13358-025-00352-x)
Supplement: Supplementary file 1 — Additional file 1. [file 13358_2025_352_MOESM1_ESM.pdf]

Supplementary material

The first record of a shortnose chimaera-like egg capsule from the Mesozoic (Late Jurassic, Switzerland)

Yang Zhao, Jordan Bestwick, Jan Fischer, Dylan Bastiaans, Merle Greif & Christian Klug

This file includes:

Character information 1

Character matrix 6

References 8

Character information

To investigate the potential relationship between the morphotypes of chondrichthyan egg capsules, our phylogenetic dataset includes 22 capsule characters from 16 taxa, modified from Fischer *et al.,* 2014. The ichnogenera *Vaillantoonia* (Brown, 1946; Kiel *et al.,* 2024), *Scyliorhinotheca* (Kiel *et al.,* 2011) and *Rajitheca* (Steininger, 1966) were added to the dataset to increase the sample size.

Our material includes five new characters, which are highlighted in bold and marked with an asterisk (*)

1. Aspect ratio (length/maximum diameter without flange and horns)

    0 ≤ 3.5 (Brown, 1946)

    1 > 3.5

2. Anterior end of the capsule

    0 pointed

    1 else

Here, the anterior end refers to the hatching side. A pointed anterior end is observed in extant chimaeroid capsules, as well as in some fossil capsules including *Palaeoxyris*, *Fayolia*, *Vetacapsula* and *Crookallia* (Dean, 1906; Fischer *et al.,* 2014; Mottequin *et al.,* 2022). The anterior end of *Laffonia* is incompletely preserved, so we tentatively code this character as uncertain for *Laffonia*.

3. Three-partition into a beak, central body, and elongated pedicle

    0 absent

    1 present

4. Length ratio of pedicle to central body

    0 ≤ 1

    1 > 1

The character is scored as inapplicable for modern elasmobranch egg capsules (Fischer *et al.,* 2014), as well as for fossil capsules *Scyliorhinotheca* (Kiel *et al.,* 2011) and *Rajitheca* (Steininger, 1966), because scoring this character is contingent on the presence of a pedicle. All modern chimaeroid capsules (Dean, 1906; Didier *et al.,* 2012) and *Palaeoxyris* (Böttcher, 2010; Fischer *et al.,* 2010) possess a pedicle longer than the length of the central body. In *Laffonia*, this character is coded with a question mark due to the incomplete preservation of the pedicle.

5. Length ratio of beak to central body

0 ≤ 1

1 > 1

6. Pedicle with a terminal filament*

0 absent

1 present

This character refers to the filament present at the end of the pedicle in extant chimaerid (Sathyanesan, 1966; Mancusi *et al.,* 2021) and callorhynchid (Dean, 1906) egg capsules.

7. Small pores on the posterior side of the capsule *

0 absent

1 present

Small respiratory pores are present along the posterior side of chimaerid and rhinochimaerid capsules (Dean, 1906; Mancusi *et al.,* 2021). This character is coded with a question mark for all fossil capsules.

8. Capsule body surface

0 smooth

1 ribbed

9. Middle ridge (dorsal keel)

0 absent

1 present

This character is scored as inapplicable for taxa lacking a ribbed body surface, as the middle ridge (dorsal keel) represents a specialized rib that is prominently positioned along the middle of the capsule. This character is present in extant chimaerid capsules and *Vetacapsula* (Dean, 1906; McGhee & Richardson, 1982; Fischer *et al.,* 2014).

10. Number of longitudinal ribs*

0 ≤ 2

1 3 - 20

2 > 20

The number of longitudinal ribs varies among the sampled taxa. Chimaerid egg capsules possess only one longitudinal rib, whereas *Laffonia* and *Crookallia* have fewer than 20 longitudinal ribs (Stainier, 1938; Fischer *et al.,* 2014; Mottequin *et al.,* 2022). Some extant and fossil scyliorhinids also exhibit fewer than 30 longitudinal ribs (Concha *et al.,* 2010; Kiel *et al.,* 2011). In contrast, *Vetacapsula* has more than 20 longitudinal ribs on the body surface (Pruvost, 1930).

11. Flange trend

0 lateral = parallel to body axis

1 twisted = helicoidal

12. Lateral flange size

0 only as a thin seam <10%

1 narrow, <50% but >10% of the maximum transversal width of the body

2 broad, >50% of maximum transversal width of the body

This character is modified from the character ‘collarette (=flange)’ in Fischer *et al.* (2014). It refers to the width ratio of the lateral flange to the capsule body and is applicable only to sampled taxa with lateral flanges (or lateral keels).

13. Number of flanges

0 ≤ 2

1 > 2

14. Scar lines close to the attachment of the flange

0 absent

1 present

15. Flange surface

0 smooth

1 ribbed or striated

16. Fine longitudinal striation on flange*

0 absent

1 present

This character is known in *Palaeoxyris*, *Fayolia*, and extant heterodontid capsules (Fischer *et al.,* 2011).

17. Attachment tendrils

0 absent

1 present

The character 'attachment tendrils' from Fischer *et al*. (2014) is now split into two: 'attachment tendrils' and 'attachment tendrils position'. This division was made to establish character polarity, as scoring tendril position requires first coding their presence. The same approach is applied to the character 'apron' below.

18. Attachment tendrils position

0 at the posterior end

1 at the anterior end

2 at the anterior and posterior ends

In *Palaeoxyris* and *Fayolia*, the tendril, when preserved, is located at the beak (anterior end), whereas in extant heterodontid capsules, the tendril is at the pointed posterior end. We therefore added a new state, ‘at the anterior end’, to reflect this difference.

19. Two pairs of horns

0 absent

1 present

20. Horn reduced*

0 absent

1 present

In orectolobid egg capsules, one horn is significantly or completely reduced, while the other is dominant and elongated (Caruso & Bor, 2007; Fischer *et al.,* 2014).

21. Apron (web at the anterior and/or posterior end)

0 absent

1 present

22. Apron position

0 at the anterior end

1 at the anterior and posterior ends

,

Character matrix

#NEXUS

BEGIN TAXA;

TITLE Capsules;

DIMENSIONS NTAX=16;

TAXLABELS

Placodermi Callorhinchidae Rhinochimaeridae Chimaeridae Heterodontidae Orectolobidae Scyliorhinidae Rajoidae Vetacapsula Crookallia Fayolia Palaeoxyris Laffonia Vaillantoonia Scyliorhinotheca Rajitheca

;

END;

BEGIN CHARACTERS;

TITLE Character_Matrix;

DIMENSIONS NCHAR=22;

FORMAT DATATYPE = STANDARD RESPECTCASE GAP = - MISSING = ? SYMBOLS = " 0 1 2 3";

CHARSTATELABELS

1 Aspect_ratio / '<= 3.5' '> 3.5',

2 Anterior_end_of_the_capsule / pointed else,

3 'Three-partition into a beak,central body, and elongated pedicle' / absent present,

4 Length_ratio_of_pedicle_to_central_body / '<= 1' '> 1',

5 Length_ratio_of_beak_to_central_body / '<= 1' '> 1',

6 Pedicle_with_a_terminal_filament / absent present,

7 Small_pores_on_the_posterior_side_of_the_capsule / absent present,

8 Capsule_body_surface / smooth _ribbed,

9 'Middle ridge (dorsal keel)' / absent present,

10 Number_of_longitudinal_ribs / '<= 2' '3-20' '> 20',

11 Flange_trend / 'lateral = parallel to body axis' 'twisted = helicoidal',

12 Lateral_flange_size / 'only as a thin seam <10%' 'narrow, <50% but >10% of the maximum transversal width of the body' 'broad, >50% of maximum transversal width of the body',

13 Number_of_flanges / '<= 2' '> 2',

14 Scar_lines_close_to_the_attachment_of_the_flange / absent present,

15 Flange_surface / smooth ribbed_or_striated,

16 Fine_longitudinal_striation_on_flange / absent present,

17 Attachment_tendrils / absent present,

18 Attachment_tendrils_position / at_the_posterior_end at_the_anterior_end at_the_anterior_and_posterior_ends,

19 Two_pairs_of_horns / absent present,

20 Horn_reduced / absent present,

21 Apron / absent present,

22 Apron_position / at_the_anterior_end at_the_anterior_and_posterior_ends ;

MATRIX

Placodermi 10?1???0--0200100-0-0-

Callorhinchidae 10110100--0200100-0-0-

Rhinochimaeridae 10110010--0200100-0-0-

Chimaeridae 1011011100000100-0-0-

Heterodontidae 010---00--1-0011100-10

Orectolobidae 010---00--00000-121111

Scyliorhinidae 010---0(0 1)0100000-121011

Rajoidae 010---00--0(0 1)000-121011

Vetacapsula 10110??1120?00??0-0-0-

Crookallia 10110??10101000-0-0-0-

Fayolia 101000?0--1-0111110-0-

Palaeoxyris 101100?0--1-1011110-0-

Laffonia ??????101010010??0-0-

Vaillantoonia 10110??0--0200100-0-0-

Scyliorhinotheca 010---?(0 1)0100000-??????

Rajitheca 010---?0--00000-??1011

;

END;

References

Böttcher, Ronald 2010. Description of the shark egg capsule *Palaeoxyris friessi* n. sp. from the Ladinian (Middle Triassic) of SW Germany and discussion of all known egg capsules from the Triassic of the Germanic Basin. *Palaeodiversity*, 3, 123-139.

Brown, Roland W. 1946. Fossil Egg Capsules of Chimaeroid Fishes. *Journal of Paleontology*, 20, 261-266.

Caruso, J. & Bor, P. H. F. 2007. Egg capsule morphology of *Parascyllium variolatum* (Duméril, 1853) (Chondrichthyes; Parascylliidae), with notes on oviposition rate in captivity. *Journal of Fish Biology*, 70, 1620-1625.

Concha, F., Bustamante, C., Oddone, M. C., Hernández, S. & Lamilla, J. 2010. Egg capsules of the dusky catshark *Bythaelurus canescens* (Carcharhiniformes, Scyliorhinidae) from the south-eastern Pacific Ocean. *Journal of Fish Biology*, 77, 963-971.

Dean, Bashford. *Chimæroid fishes and their development.* Carnegie Institution of Washington, Washington, D.C., 1906.

Didier, Dominique A., Kemper, Jenny M. & Ebert, David A. Phylogeny, Biology and Classification of Extant Holocephalans. in *Biology of Sharks and Their Relatives,* edited by J. C. Carrier, J. A. Musick & M. R. Heithaus. CRC Press2012, Vol. 2, pp. 97–122.

Fischer, Jan, Axsmith, Brian J. & Ash, Sidney R. 2010. First unequivocal record of the hybodont shark egg capsule *Palaeoxyris* in the Mesozoic of North America. *Neues Jahrbuch für Geologie und Paläontologie - Abhandlungen*, 255, 327-344.

Fischer, Jan, Licht, Martin, Kriwet, Jürgen, Schneider, Jörg W., Buchwitz, Michael & Bartsch, Peter 2014. Egg capsule morphology provides new information about the interrelationships of chondrichthyan fishes. *Journal of Systematic Palaeontology*, 12, 389-399.

Fischer, Jan, Voigt, Sebastian, Schneider, Jörg W., Buchwitz, Michael & Voigt, Silke 2011. A selachian freshwater fauna from the Triassic of Kyrgyzstan and its implication for Mesozoic shark nurseries. *Journal of Vertebrate Paleontology*, 31, 937-953.

Kiel, Steffen, Peckmann, Jörn & Simon, Klaus 2011. Catshark egg capsules from a Late Eocene deep-water methane-seep deposit in western Washington State, USA. *Acta Palaeontologica Polonica*, 58, 77-84.

Kiel, Steffen, Stevens, Thomas & Goedert, James L. 2024. An Oligocene chimaeroid egg capsule from western Washington State, USA, and priority of *Vaillantoonia* Meunier, 1891. *Journal of Paleontology,* 1-4.

Mancusi, C., Massi, D., Baino, R., Cariani, A., Crobe, V., Ebert, D. A., Ferrari, A., Gordon, C. A., Hoff, G. R., Iglesias, S. P., Titone, A. & Serena, F. 2021. An identification key for Chondrichthyes egg cases of the Mediterranean and Black Sea. *The European Zoological Journal*, 88, 436-448.

McGhee, George R. & Richardson, Eugene S. 1982. First occurrence of the problematical fossil *Vetacapsula* in North America. *Journal of Paleontology*, 56, 1295-1296.

Mottequin, Bernard, Fischer, Jan, Goolaerts, Stijn & Olive, Sébastien 2022. Revisiting the chondrichthyan egg capsules inventory from the Pennsylvanian (Carboniferous) of Belgium: new data and perspectives. *The Science of Nature*, 109, 39.

Pruvost, P. 1930. La faune continentale du terrain houiller de la Belgique. *Mémoires du Musée royal d'histoire naturelle de Belgique*, 44, 103-280.

Sathyanesan, A. G. 1966. Egg-Laying of the Chimaeroid Fish *Hydrolagus colliei*. *Copeia,* 132-134.

Stainier, Xavier 1938. *Belinurus* et *Vetacapsula* nouveaux de Houiller belge. *Bulletin du Musée Royal d'Histoire Naturelle de Belgique*, 14, 1-13.

Steininger, F. V. 1966. Zur Kenntnis fossiler Euselachier-Eikapseln aus dem Ober-Oligozän von Mitteleuropa. *Mitteilungen der Bayerischen Staatssammlung für Paläontologie und histor. Geologie*, 6, 37-49.
